# Hepatic Overexpression of Soluble Urokinase Receptor (uPAR) Suppresses Diet-Induced Atherosclerosis in Low-Density Lipoprotein Receptor-Deficient (LDLR^-/-^) Mice

**DOI:** 10.1371/journal.pone.0131854

**Published:** 2015-08-27

**Authors:** Jan Larmann, Kerstin Jurk, Henrike Janssen, Martin Müller, Christine Herzog, Anika Lorenz, Martina Schmitz, Jerzy-Roch Nofer, Gregor Theilmeier

**Affiliations:** 1 Department of Anesthesiology and Intensive Care Medicine, Hannover Medical School, Hannover, Germany; 2 Department of Anesthesiology University Hospital Heidelberg, Heidelberg, Germany; 3 Center for Thrombosis and Hemostasis (CTH), University Medical Center, Mainz, Germany; 4 Institute for Anatomy, University of Münster, Münster, Germany; 5 Center for Laboratory Medicine, University Hospital Münster, Münster, Germany; 6 Department of Health Services Sciences, Faculty of Medicine and Health Sciences, University of Oldenburg, Oldenburg, Germany; University Heart Center Freiburg, GERMANY

## Abstract

**Objective:**

Atherosclerosis, a chronic inflammatory disease, arises from metabolic disorders and is driven by inappropriate recruitment and proliferation of monocytes / macrophages and vascular smooth-muscle-cells. The receptor for the urokinase-type plasminogen activator (uPAR, Plaur) regulates the proteolytic activation of plasminogen. It is also a coactivator of integrins and facilitates leukocyte-endothelial interactions and vascular smooth-muscle-cell migration. The role of uPAR in atherogenesis remains elusive.

**Methods and Results:**

We generated C57Bl6/J low-density lipoprotein receptor (LDL) and uPAR double knockout (uPAR^-/-^/LDLR^-/-^) mice to test the role of uPAR in two distinct atherosclerosis models. In LDLR^-/-^ mice, hepatic overexpression following hydrodynamic transfection of soluble uPAR that competes with endogenous membrane-bound uPAR was performed as an interventional strategy. Aortic root atherosclerotic lesions induced by feeding a high-fat diet were smaller and comprised less macrophages and vascular smooth-muscle-cells in double knockout mice and animals overexpressing soluble uPAR when compared to controls. In contrast, lesion size, lipid-, macrophage-, and vascular smooth muscle cell content of guide-wire-induced intima lesions in the carotid artery were not affected by uPAR deficiency. Adhesion of uPAR^-/-^-macrophages to TNFα-stimulated endothelial cells was decreased *in vitro* accompanied by reduced VCAM-1 expression on primary endothelial cells. Hepatic overexpression of soluble full-length murine uPAR in LDLR^-/-^ mice led to a reduction of diet-induced atherosclerotic lesion formation and monocyte recruitment into plaques. *Ex vivo* incubation with soluble uPAR protein also inhibited adhesion of macrophages to TNFα-stimulated endothelial cells *in vitro*.

**Conclusion:**

uPAR-deficiency as well as competitive soluble uPAR reduced diet-promoted but not guide-wire induced atherosclerotic lesions in mice by preventing monocyte recruitment and vascular smooth-muscle-cell infiltration. Soluble uPAR may represent a therapeutic tool for the modulation of hyperlipidemia-associated atherosclerotic lesion formation.

## Introduction

Atherosclerosis is an inflammatory disease of the vessel wall characterized by monocyte recruitment to lesion-prone sites and their dedifferentiation into foam cells.[1, 2] Macrophages and vascular smooth muscle cells (VSMC) in the lesion release inflammatory mediators and proteolytic activity driving lesion progression.[3] The receptor for the plasminogen activator of the urokinase type (CD87, Plaur, uPAR), a GPI-anchored glycoprotein, regulates membrane-bound proteolytic activity of uPA by increasing its efficiency for plasminogen cleavage.[4] Proteolytic activity is thought to be associated with lesional cell migration and, through extracellular matrix breakdown, reduced plaque stability.[5, 6] Hence, uPA overexpressing macrophages stimulate atherosclerosis.[7] uPAR is also a cofactor for beta-integrin-activation and thus facilitates cell-cell interactions[4, 8] as well as transendothelial recruitment of neutrophils[9] and macrophages.[10] uPAR is expressed in human atherosclerotic lesions, and its expression is associated with lesion progression.[11–14] However, its role during atherogenesis, the underlying mechanisms thereof and its potential for therapeutic interventions remain largely elusive. Using the low-density lipoprotein-receptor deficiency (LDLR^-/-^)-[15] and Western-diet-induced model of dyslipidemia and atherosclerosis, we analysed the effect of uPAR-deficiency on atherogenesis by comparing uPAR’s role for i) diet-induced spontaneous aortic root atherosclerosis and ii) guide-wire injury (GWI) induced intimal hyperplasia in the carotid artery. In addition, in a therapeutic approach, we investigated the impact of hepatic overexpression of soluble uPAR (suPAR) as competitive inhibitor of endogenous membrane bound uPAR in diet-induced atherosclerosis in LDL-receptor-deficient mice.

## Materials and Methods

### Animals

uPAR-deficient (*uPAR* ^-/-^) mice[16] were crossbred with LDLR-deficient (*Ldlr* ^*-/-*^) mice (Jackson Laboratories, Bar Harbor, Maine) in the C57Bl/6J-background (>F10). The first double heterozygous offspring were bred to obtain uPAR^-/-^/LDLR^-/-^ and uPAR^+/+^/LDLR^-/-^ control mice with identical genetic background, which were perpetuated separately for 3–4 generations. Genotypes were determined by polymerase chain reaction. Animals were fed a cholate-free high-fat diet (1.4% cholesterol, 12% cocoabutter, 3% oil, TD95046-modified, Harlan, Germany). All mice were housed under pathogen-free conditions. All animal experiments were performed in accordance with protocols approved by the local animal research committee (Bezirksregierung Münster, Az: 23.0835.1.0 (G 62/2000)) and in accordance with the Guide for the Care and Use of Laboratory Animals published by the National Academy of Science and all efforts were made to minimize suffering.

### Diet-induced atherosclerosis

For the diet induced atherosclerosis model 64 age- and gender-matched uPAR^-/-^/LDLR^-/-^ and uPAR^+/+^/LDLR^-/-^ mice (6±0.5 weeks; 18±3.4 g) were maintained on the atherogenic diet. After 10 weeks on diet, animals were anesthetized with 1.5 Vol% Isoflurane in 100% oxygen. Following a laparotomy the inferior caval vein was cannulated. Mice were exsanguinated and perfused from a left ventricular puncture using 0.9% saline. The base of the heart with the aortic root were harvested.[17]

### Guide-wire injury induced intimal hyperplasia

After 6 weeks on diet we performed a GWI procedure as described previously[18, 19] in a subgroup of 20 age- and gender-matched mice to induce intimal hyperplasia of the left carotid artery. Endothelial denudation was achieved by introducing a 21-gauge guide-wire (Arrow International, Reading, PA) three times from the left external carotid artery into the common carotid artery as previously described.[18] For each animal the right carotid artery served as sham control. The vessel was dissected likewise but no GWI was performed. Carotid arteries were collected 4 weeks later.[17] 7μm thick cryosections were made and approximately 100 sections per mouse were analyzed through the whole injured artery.

### Plasma lipid and lipoprotein analyses

Blood was drawn from the retro bulbar plexus under isoflurane anesthesia at 0, 3, 6, and 9 weeks to determine lipoprotein plasma levels. We measured plasma total cholesterol, HDL cholesterol, and triglycerides photometrically using commercially available kits (Roche, Mannheim, Germany). LDL cholesterol was calculated by the Friedewald formula.

### Atherosclerotic lesion size and composition

Collected tissue was cryoembedded and stored at -80°C. Serial 7 μm sections were prepared and stained for oil red-O to identify lipids. To control for gender bias, animals were also analyzed after stratification into male and female animals. On adjacent sections, macrophages were stained (rat anti-mouse Mac-3 (clone M3/84), BD Pharmingen, Heidelberg Germany or CD68 (rat anti-mouse antibody (clone FA-11), Serotec, Düsseldorf, Germany)). VSMC (clone: 1A4, Sigma, Taufkirchen, Germany) were stained using a mouse on mouse protocol. Secondary antibodies for detection were: Cy3-coupled goat anti-mouse (Jackson ImmunoResearch, West Grove, USA) and goat anti-rat IgG-Cy5 (Abcam, Cambridge, UK). A peroxidase coupled secondary antibody and DAB (Vectorstain, DAKO, Hamburg, Germany) as substrate was used for detection of rat anti-mouse Mac-3. Double immunohistochemistry for CD68 and Proliferating Cell Nuclear Antigen (PCNA) was performed in aortic sinus lesions using a polyclonal anti-PCNA antibody (Abcam, Cambridge, United Kingdom). To assess macrophage apoptosis CD68/ cleaved caspase 3 (Cell Signaling, Danvers, MA) double staining was performed. In carotid lesions we prepared a VSMC and PCNA double immunohistochemestry staining. Images were acquired using an Olympus IX81 (Olympus, Hamburg, Germany) or Eclipse TE300 (Nikon, Düsseldorf, Germany) microscope. A Retiga EXi camera (QImaging, Surrey, British Columbia, Canada) and QCapture Pro (version 6.0.0.412, QImaging) or analysis (SIS, Münster, Germany) software were used to capture and analyze images.

### Recombinant protein production and hepatic overexpression of soluble full-length murine uPAR

Full-length uPAR was cloned in frame into the pPICZA vector, expressed in a Pichia pastoris system and purified using a c-myc-tag (Life Technologies, Darmstadt, Germany). For *in vivo* gene delivery the cDNA encoding full-length murine uPAR was subcloned into an expression vector containing apoAI promotor and apoE enhancers.[20] For hydrodynamic transfection[21] 50μg of plasmid DNA was dissolved in 0.9% saline and rapidly injected over 10sec. via a 26G tail vein catheter into 16 age and gender matched LDLR^-/-^ mice (12.2±0.9 weeks, 23.6±2 g). Subsequently, the animals were started on the atherogenic diet for 10 weeks. Upon sacrifice, transfection efficiency was proven by immunnohistochemstry of liver samples for uPAR.

### Harvesting of peritoneal macrophages and cardiac endothelial cells

Mononuclear cells were elicited by intraperitoneal injection of 500μl 4% Brewer’s-Thioglycollate-Medium in C57Bl/6J mice as described.[10] Animals were sacrificed on day 3 and peritoneal lavage was collected in ice-cold Hanks buffered salt solution (HBSS) free of Ca2+/Mg2+. Leukocytes were counted and resuspended at 10^6^/mL in HBSS. For isolation of primary murine EC, uPAR^-/-^ and WT animals were exsanguinated in isoflurane anesthesia and perfused from a left ventricular puncture using 0.9% saline. Hearts were collected and the myocardium was digested by collagenase. EC were harvested by magnetic cell sorting (MACS, Miltenyi Biotec, Auburn, CA) using anti-CD31 antibody-coated magnetic beads according to the manufacturer’s instructions.

### Cell culture

Cell culture materials were purchased from PAA (Coelbe, Germany). Immortalized murine EC (f.End5)[22, 23] were grown in Dulbecco’s modified Eagle medium, macrophages were cultured in RPMI-1640. Primary CD31-positive EC were plated in gelatin-coated culture flaskes and grown in complete Endothelial Basal Medium-2 (EBM-2) (Lonza, Verviers, Belgien). Cells were split at a 1:3 ratio. The purity of ECs was confirmed by immunofluorescent CD-31 labeling. All media were supplemented with 10% fetal bovine serum, L-glutamine, penicillin and streptomycin.

### Mononuclear cell adhesion

We studied endothelial adhesiveness for mouse peritoneal macrophages from WT and uPAR^-/-^ mice in a parallel-plate flow-chamber model.[18] Thioglycollate-elicited peritoneal macrophages were labelled with Cell Tracker Green (Invitrogen, Karlsruhe, Germany) according to the manufacturers instructions. Cells were perfused across fEnd.5 at 100s^-1^. We activated the endothelium over 12 hours using 10 ng/ml tumor necrosis factor-alpha (TNFα) (Biosource, Camarillo, CA). TNFα activation was performed in the presence or absence of 100μg/ml suPAR to test uPARs therapeutic potential. We quantified firm adhesion on pictures taken from 15 high-power fields captured after 3 minutes of cell perfusion followed by 3 minutes of buffer wash using a confocal microscope (UltraView, Perkin Elmer, Juügesheim, Germany).

### VSMC migration

VSMC were harvested from WT and uPAR^-/-^ mice using an outgrowth assay.[24] For migration assays, VSMC in DMEM supplemented with 10% FBS were seeded in six-well plates. Sub-confluent cells were starved for 72 hours in DMEM supplemented with 0.2% FBS. Cell migration was evaluated using a scratch wound assay.[24] The cell monolayer was scratched using a pipet tip. The remaining cells were washed twice with 0.2% FBS. After 24 hours closure of the scratch was assessed as the distance migrated on 10 high-power fields (hpf) along the scraped line.

### Real-time RT-PCR

Total RNA was extracted from primary ECs by TRItidy reagent (Applichem, Darmstadt, Germany). All reagents and primer were purchased from Eurogentec (Cologne, Germany). cDNA was obtained by reverse transcription of 500ng RNA. The primer sequences were as follows: mouse ICAM-1 forward, 5’-TCT-AAT-GTC-TCC-GAG-GCC-AGG-A-3’, reverse, 5’-GTT-ACT-TGG-CTC-CCT-TCC-GAG-A-3’; mouse VCAM-1 forward, 5’-ACT-CTA-CTG-CGC-ATC-TTC-CTT-GGT-3’, reverse, 5’-AAT-GCA-TGG-CTT-GGT-TTG-TGG-AGG-3’, mouse HPRT forward, 5’-TGA-TCA-GTC-AAC-GGG-GGA-CAT-A-3’, reverse, 5’-GCC-TGT-ATC-CAA-CAC-TTC-GAG-A-3’. Samples were run in duplicates on a real time PCR cycler (Rotorgene 3000, Corbett Life Science, Hilden, Germany). Signals were generated by MESA-GREEN incorporation into the amplified DNA and normalized to GAPDH expression.

### Statistics

Data were analyzed using GraphPad Prism 5.0b (GraphPad Software, San Diego CA). Groups were compared by Mann–Whitney U test or Kruskal-Wallis test with Dunn’s post-test. Spearman-Rho test calculated the correlation between lesion size and macrophage content. Data are presented as mean±SEM. P<0.05 was considered significant.

## Results

### uPAR deficiency prevents macrophage recruitment and protects against diet-induced atherosclerosis

**Fig 1:**
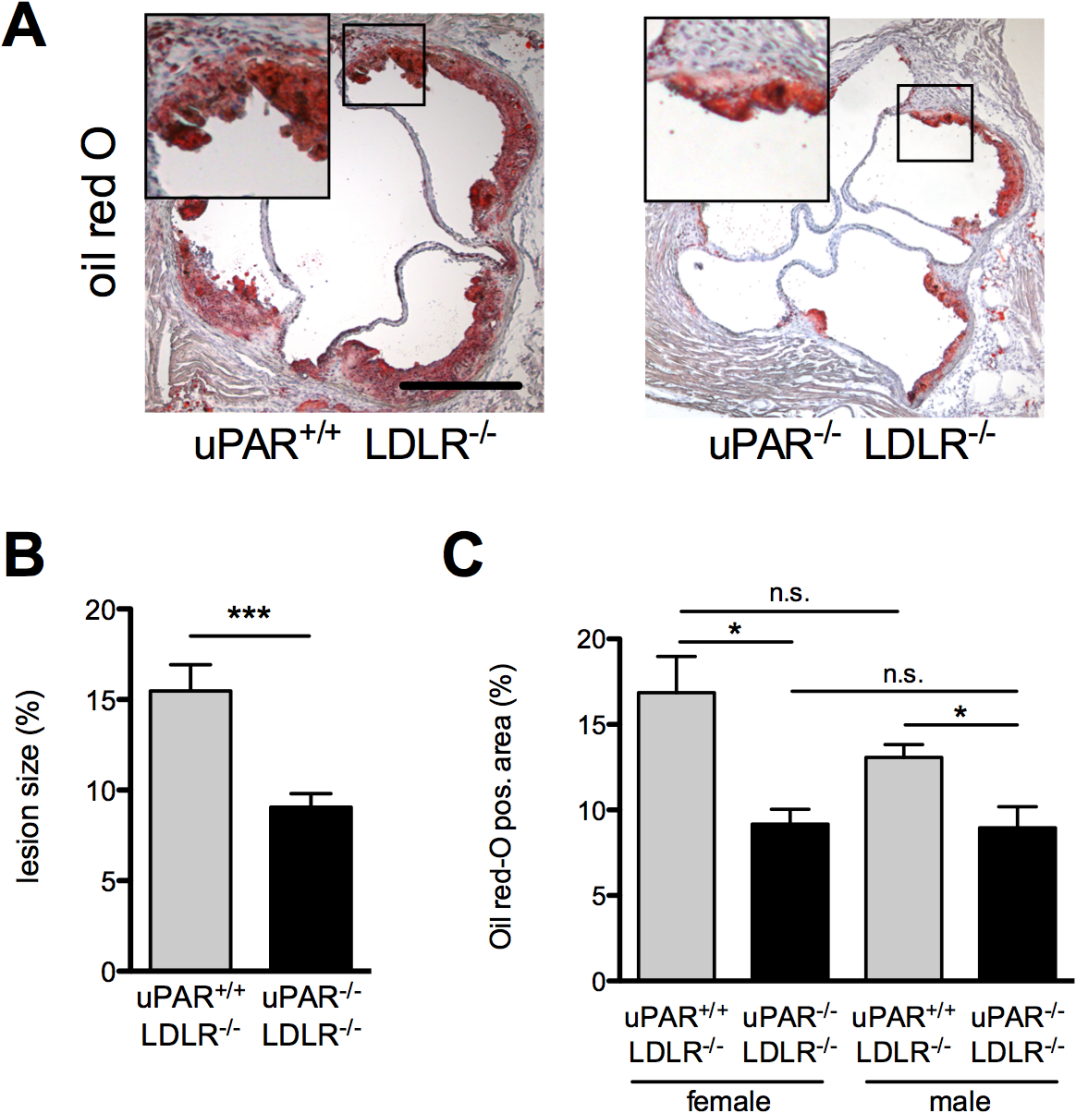
uPAR-deficiency reduces atherosclerotic lesion size in LDLR^-/-^ mice. (A) Oil red-O staining of aortic valve cryosections. (B) Atherosclerotic lesion size was assessed as % total aortic sinus lumen area occupied. (C) Quantification of lesion size in uPAR^-/-^ LDLR^-/-^ (n = 12) and uPAR^+/+^ LDLR^-/-^ (n = 13) mice was performed on oil red-O stainings. Lesion development between male and female mice did neither differ in uPAR^-/-^ LDLR^-/-^ (female: n = 6; male; n = 6) nor in uPAR^+/+^ LDLR^-/-^ mice (p = 0.229; female: n = 7; male: n = 6). Mean±SEM. *P<0.05, ***P<0.001.

No differences were observed for body weight, fertility, general health status, or dyslipidemia (Table 1) between uPAR^-/-^/LDLR^-/-^ and uPAR^+/+^/LDLR^-/-^ mice receiving the high-fat diet. After 10 weeks on this atherogenic diet gender matched uPAR^-/-^/LDLR^-/-^ animals had developed smaller aortic root atherosclerotic lesions than control mice (Fig 1A and 1B). This effect remained robust when we stratified animals for gender (Fig 1C). Lesion development in male and female uPAR^+/+^ LDLR^-/-^ mice did not differ significantly (p = 0.229).

**Table 1:** Lipoprotein profiles. The cholesterol rich diet induced hyperlipidemia in uPAR wild-type and knockout mice in the LDLR^-/-^ background. Within three weeks total and LDL-cholesterol were significantly increased along with triglycerides. No uPAR-dependent differences between the cohorts were observed. HDL-cholesterol increased along with the atherogenic lipoproteins and triglycerides.

Lipoprotein	Week	uPAR^-/-^ / LDLR^-/-^	uPAR^+/+^ / LDLR^-/-^	P value
Total cholesterol, mg/dl	0	129 ± 3.2	165 ± 4.5	n.s.
	3	782.5 ± 52.8	914 ± 67.2	n.s.
	6	1198 ± 395.8	1143 ± 65.0	n.s.
	9	1012 ± 60.5	1156 ± 52.5	n.s.
				
HDL cholesterol, mg/dl	0	6.6 ± 4.4	84.2 ± 3.6	n.s.
	3	242.3 ± 13.5	279.4 ± 13.6	n.s.
	6	287 ± 44.4	292 ± 8.1	n.s.
	9	269 ± 7.7	306 ± 8.7	n.s.
				
LDL cholesterol, mg/dl	0	44.6 ± 4	63.2 ± 3.2	n.s.
	3	500.4 ± 41.3	594.9 ± 53.5	n.s.
	6	841 ± 319.6	777 ± 50.1	n.s.
	9	693 ± 49.5	804 ± 42.3	n.s.
				
Triglycerides, mg/dl	0	74.8 ± 9.2	88.3 ± 8.3	n.s.
	3	199.4 ± 20	199.9 ± 12.2	n.s.
	6	347 ± 164.9	365 ± 55.7	n.s.
	9	250 ± 42.1	232 ± 24.3	n.s.
				

Mean±SEM, n = 10. n.s. = not significant, HDL: high-density lipoprotein, LDL: low-density lipoprotein.

**Fig 2:**
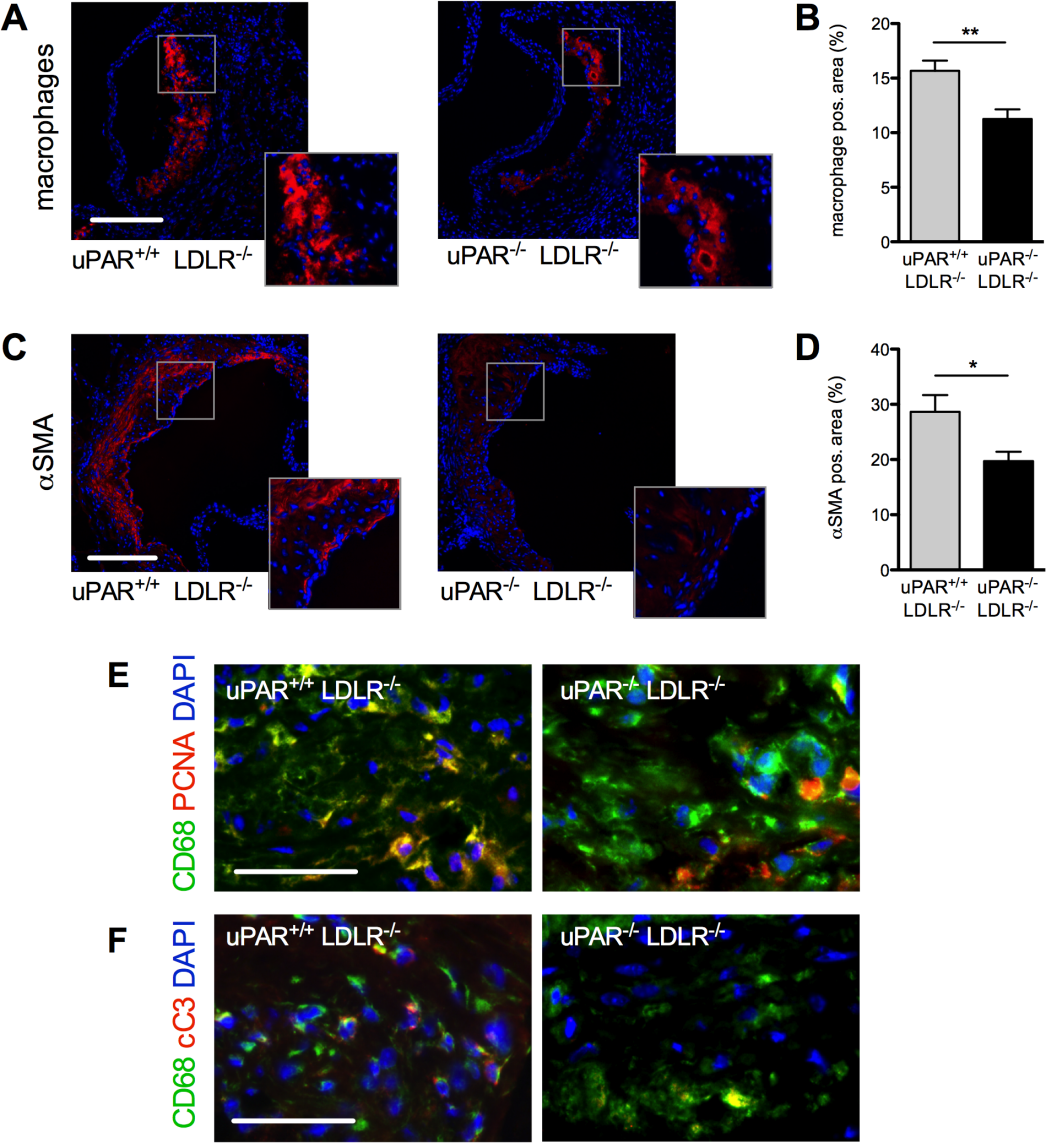
Macrophage and VSMC accumulation is reduced in uPAR^-/-^/LDLR^-/-^. (A) Representative Mac-3 immunostaining and (B) %-Mac-3-positive lesion per lesion area. (C) Representative anti-SMC immunostaining and (D) % anti-SMC-positive lesion per lesion area. (E) PCNA/CD68 double staining excludes differences in macrophage proliferation in the lesions. (F) Macrophage apoptosis was not responsible for the differences in macrophage content as evidenced by cleaved caspase 3 staining. Mean±SEM, n = 8. *P<0.05, **P<0.01, ***P<0.001. A/C: Bar = 250 μm, E/F Bar = 100μm αSMA: alpha smooth-muscle-actin, PCNA: Proliferating-Cell-Nuclear-Antigen, cC3: cleaved caspase-3, DAPI: 4′,6-Diamidin-2-phenylindol.

In this diet-induced atherosclerosis model, reduced plaque size was accompanied by a reduction in macrophage- and aSMA-positive area inside the lesions (Fig 2). Plaque size and macrophage content correlated significantly (uPAR^-/-^/LDLR^-/-^: R = 0.825, P = 0.022; uPAR^+/+^/LDLR^-/-^: R = 0.943, P = 0.0014), indicating that uPAR may have driven lesion formation via monocyte recruitment. In addition, no relevant differences between the groups were observed on CD68/PCNA or CD68/cC3 double-immunohistochemical stainings (Fig 2). Thus, neither reduced macrophage proliferation nor enhanced apoptosis contributed significantly to the observed difference in macrophage content.

### Guide-wire-induced carotid artery intimal hyperplasia was not affected by uPAR deficiency

**Fig 3:**
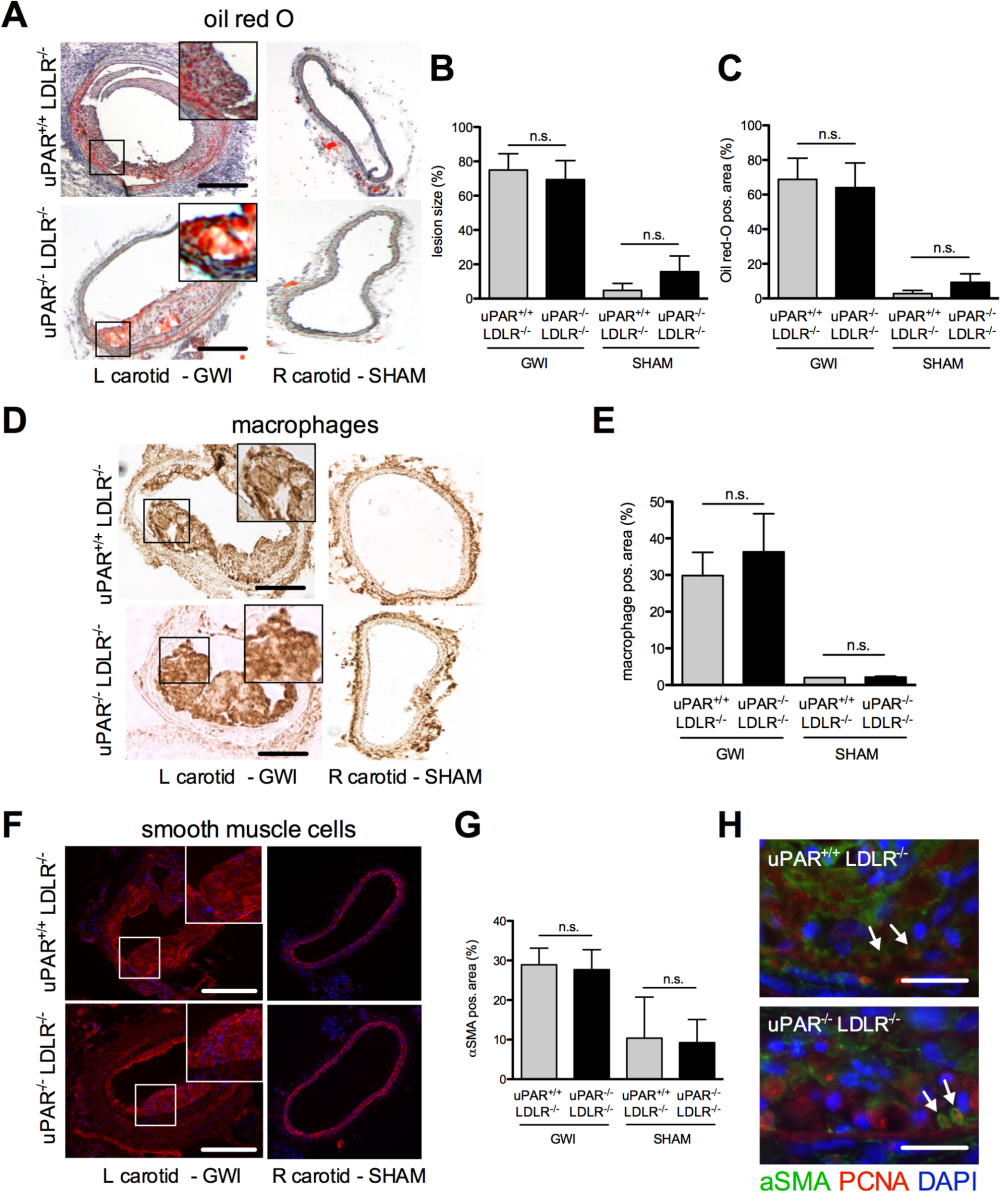
Guide-wire injury induced intimal hyperplasia. Mice were subjected to guide wire injury of the internal carotid artery after hypercholesterolemia had been induced. (A) Representative Micrographs of carotid artery lesions in the injured and the contralateral artery. GWI induced concentric lesions did not differ in (B) size or (C) lipid content. (D) Macrophage recruitment did occur, but (E) differences between uPAR-deficient and uPAR-wild-type lesions were not observed in this model. Additionally, no differences between uPAR^+/+^ and uPAR^-/-^ animals were observable with respect to (F-G) VSMC content of the lesions. No relevant lesion formation was observed in the contralateral sham vessels. (H) Proliferating cells in atherosclerotic lesions are mainly non-VSMC. The amount of proliferating VSMC appears similar for the two genotypes. A, D, F: Bar = 250μm, H: Bar: 50μm, mean±SEM, n = 8. n.s.: not significant, αSMA: alpha smooth-muscle-actin, DAPI: 4′,6-Diamidin-2-phenylindol, GWI: guide-wire injury, L: left, R: right, PCNA: Proliferating Cell Nuclear Antigen.

Within four weeks of endothelial denudation mice developed complex atherosclerotic plaques in the left carotid artery that was subjected to GWI. Lesions in sham vessels were small, did not differ between genotypes, and could be neglected compared to lesions after GWI. (Fig 3). Also, in this model no uPAR dependent differences were detected with respect to lipid-, macrophage-, or VSMC-content (Fig 3). Only very few VSMCs stained positive for PCNA and no major differences were observed between uPAR wild-type and knockout mice (Fig 3H).

### uPAR-/—macrophages display reduced adhesiveness to EC

**Fig 4:**
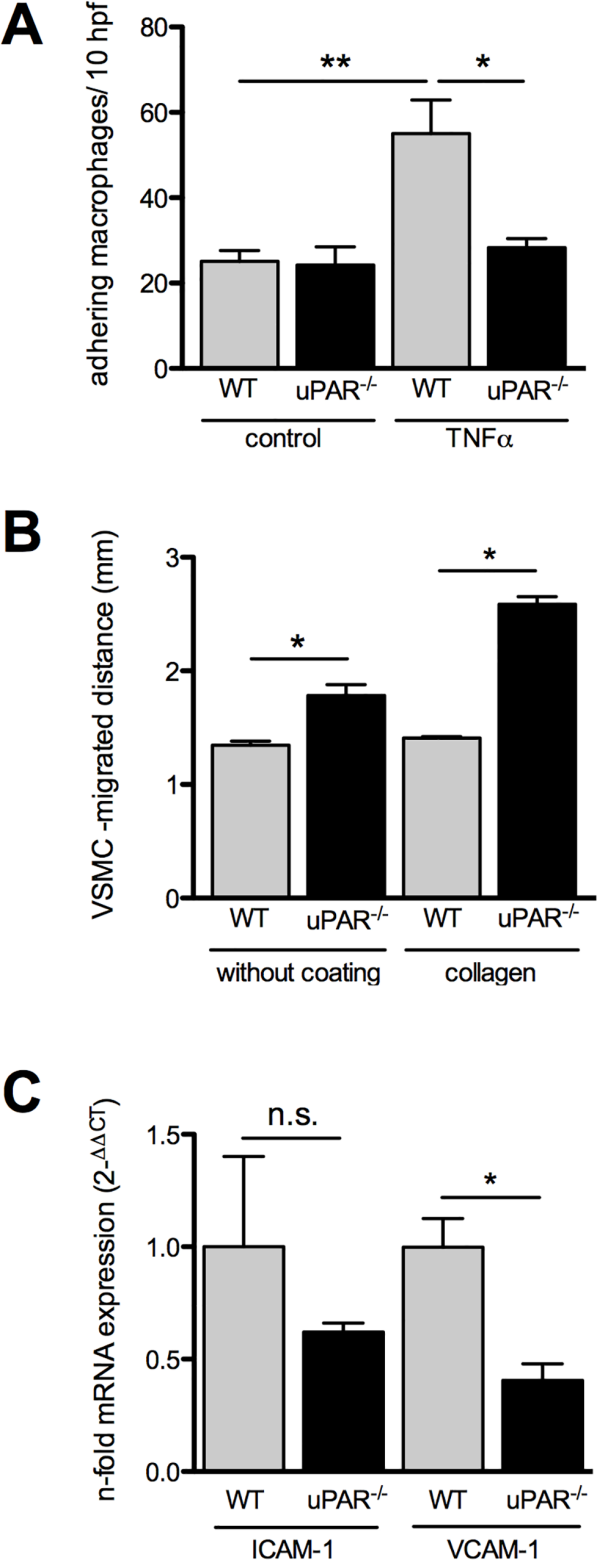
Macrophage adhesion and VSMC migration are dependent on uPAR. Macrophages were subjected to a low shear adhesion assay on murine EC. A. Macrophage adhesion to resting and activated endothelium in vitro is reduced if macrophages are harvested from uPAR-deficient animals. B. The capability of uPAR^-/-^-VSMC to migrate into a scratch was assessed in vitro and revealed increase in the migrated distance. C. mRNA expression of VCAM but not ICAM in primary murine ECs is reduced in uPAR deficient animals (n = 6) compared to controls. Mean±SEM, n = 5–8. *P<0.05, **P<0.01. Bar = 250 μm. hpf: high-power fields, WT: wild-type, TNFα: tumor necrosis factor-alpha, VSMC: vascular smooth muscle cells.

We assessed monocyte adhesion and migration as prerequisite for recruitment into atherosclerotic plaques in *ex vivo* assays. In the parallel-plate flow chamber assay macrophages, isolated from wild-type mice, showed increased adhesion when EC were stimulated with TNFα. This effect was abolished when uPAR^-/-^-macrophages were tested (Fig 4).

### VSMC migration on collagen is governed by uPAR

uPAR deficiency enhanced VSMC migration in the scratch assay regardless whether collagen coated or uncoated dishes were used. The difference on collagen-coated dishes was more prominent (Fig 4B).

### uPAR deficiency affects VCAM-1 expression

We tested whether uPAR deficiency would affect the expression of the canonical adhesion molecules ICAM-1 and VCAM-1 that both play a critical role for macrophage adhesion and VSMC migration. VCAM-1 mRNA expression was lower in EC from uPAR knock out mice compared to wild-type cells. For ICAM-1 expression we found a numerical difference that was however not statistically significant in our experiments (Fig 4C).

### Overexpression of soluble full-length murine uPAR reduced diet induced atherosclerotic lesion formation

**Fig 5:**
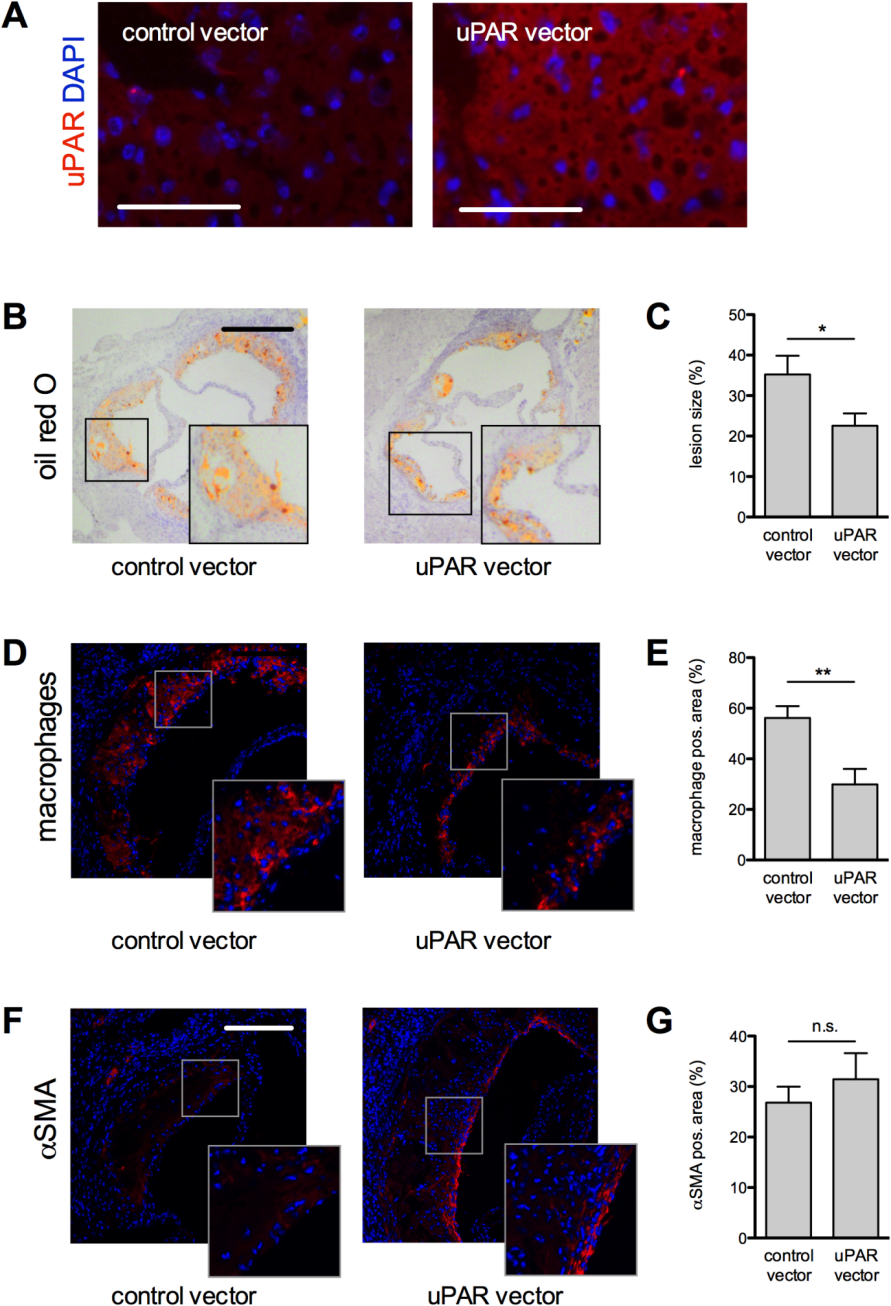
Hepatic overexpression of soluble full-length uPAR inhibits atherosclerotic lesion development and macrophage accumulation in LDLR^-/-^ mice. A, Immunostaining for the c-myc-tag revealed expression of soluble uPAR in the livers of mice after hydrodynamic transfection. Bar = 100 μm B. Oil red-O staining of aortic valve cryosections. Bar = 250 μm C. Atherosclerotic lesion size was assessed as % total aortic sinus lumen area occupied. D, Representative CD68 immunostaining and E. % CD68-positive lesion area. F, Representative anti-αSMC immunostaining and G. % anti-αSMA-positive lesion area. Bar = 250 μm, Mean±SEM, n = 6/8. *P<0.05, **P<0.01, ***P<0.001. αSMA: alpha smooth-muscle-actin, DAPI: 4′,6-Diamidin-2-phenylindol

Hepatic over-expression of uPAR in mice that underwent hydrodynamic transfection with cDNA encoding full-length murine uPAR was proven by immunohistochemistry (Fig 5A). suPAR led to a significant reduction in diet-induced aortic root atherosclerotic lesion size (Fig 5B and Fig 5C) indicating that suPAR competes endogenous membrane bound uPAR. Lesional macrophage- but not VSMC-content was reduced in mice overexpressing suPAR as compared to mice treated with control vector (Fig 5). Next we sought to test the relevance of suPAR for monocyte adhesion in vitro.

### suPAR prevents monocyte adhesion to activated endothelium

**Fig 6:**
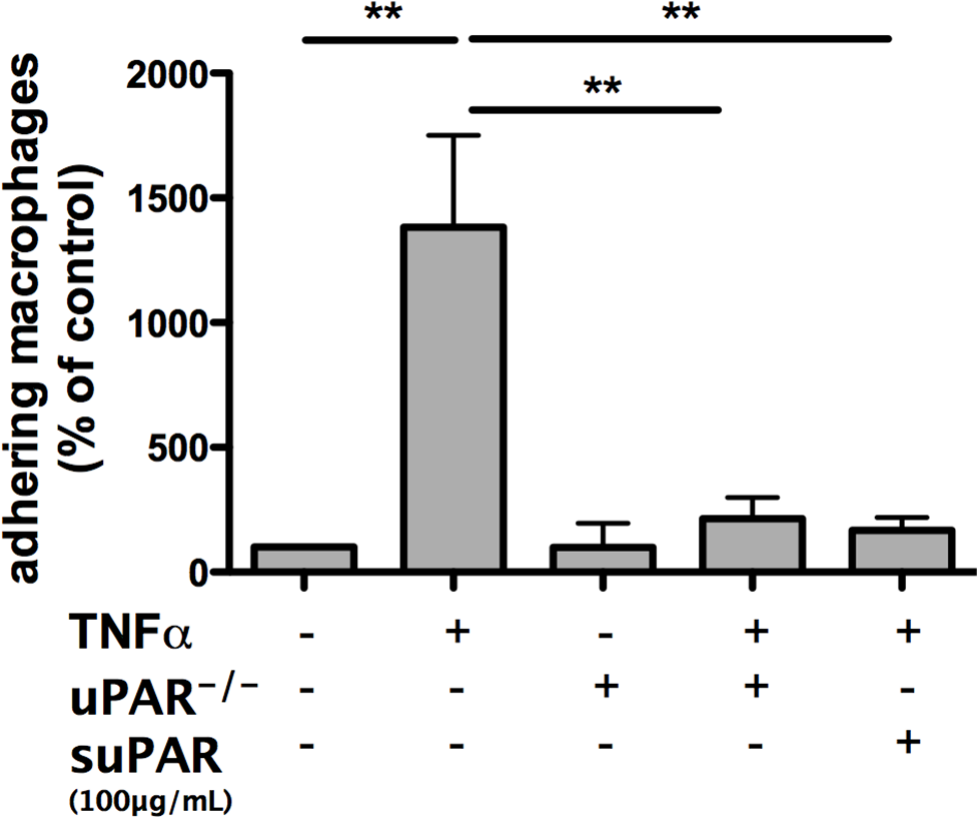
Soluble uPAR reduces macrophage adhesion to TNFα activated murine endothelial cells. Wild-type macrophage adhesion to resting and activated endothelium is significantly reduced in the presence of soluble uPAR in vitro. Mean±SEM, n = 4/5. **P<0.01. TNFα: tumor necrosis factor-alpha, suPAR: soluble urokinase-type plasminogen activator receptor.

Macrophages from wild-type mice did not show increased adhesion to TNFα stimulated EC when EC were activated in the presence of suPAR. The magnitude of the effect mediated by suPAR was comparable to the adhesion deficit observed for macrophages from uPAR^-/-^-animals (Fig 6).

## Discussion

We here demonstrate for the first time that genetic deficiency for uPAR prevents monocyte recruitment and reduces development of diet induced aortic arch atherosclerotic lesions in Western-diet fed LDLR^-/-^ mice. In addition, we established a gene therapeutic strategy of hepatic overexpression of soluble full-length murine uPAR as an effective intervention to reduce lesional macrophage content and attenuate plaque development.

Previous studies demonstrated an association of enhanced uPAR expression in atherosclerotic lesions with the severity of atherosclerosis[11] and with the frequency of plaque rupture in symptomatic carotid atherosclerosis.[12] In a mouse atherosclerosis model uPAR expression in macrophages is associated with lesion progression.[13] We could recently demonstrate that uPAR induces differentiation of mesenchymal stems cells into osteoblasts and thus promotes vascular calcification.[14] Another important contribution to the field comes from Farris and colleagues. They report that macrophage overexpression of urokinase plasminogen activator (uPA) aggravates atherosclerosis independently of uPAR.[7] By oil red-O en face staining of the whole aorta, they also demonstrate that uPAR knockout mice develop smaller atherosclerotic lesions. The effect demonstrated in their paper is smaller than the one found in our study. This can easily be explained by the different experimental settings. In their experiments a different diet was used, mice were younger when started on the atherogenic diet, and they were fed the diet twice as long. However, we are also the first to report that uPAR promotes a direct effect on monocyte adhesion and recruitment. In addition, the therapeutic potential of soluble full-length murine uPAR for prevention on lesion development has not been reported previously. To the best of our knowledge an in depth analysis of the direct involvement of uPAR in atherosclerotic lesion formation and the therapeutic potential of targeting uPAR have not been reported to date.

uPAR^-/-^/LDLR^-/-^ mice developed smaller aortic root atherosclerotic plaques than their wild-type littermates. Previous studies investigating vascular lesions in uPAR^-/-^ animals did not report lipoprotein plasma levels.[13, 14] In our model, we could not identify alterations in the lipoprotein plasma levels that would explain the observed differences in lesions size. However, protection from lesion development was accompanied by reduced macrophage content—a phenomenon that could be explained by alterations in recruitment, proliferation, or apoptosis. In vivo, apoptosis and proliferation of macrophages in atherosclerotic plaques were comparable between groups. Our evidence for a contribution of uPAR-dependent monocyte-endothelial cell interaction[9] to macrophage accumulation has been additionally supported using an in vitro approach. In contrast to macrophages isolated from wild-type mice uPAR^-/^-macrophages did not show increased adhesion to TNFα-stimulated endothelium. These findings suggest that uPAR-deficiency slows lesion progression by reducing monocyte adhesion and recruitment.

In vitro studies recapitulated the previously reported uPAR-dependent VSMC- migration on collagen.[25] We expected that this phenomenon would affect VSMC content in our in vivo model. Several previous studies describe alterations of VSMC migration in vitro,[25, 26] in vessel explants[25] or in a rat vessel injury model[27] that are mediated or associated with uPAR expression. However, to the best of our knowledge these observations have never been tested in the light of atherosclerotic lesion development. Our observations regarding enhanced VSMC migration of uPAR-deficient cells in vitro and VSMC content in uPAR-deficient mice are controversial. The reduction of VSMC content, observed in aortic sinus lesions of uPAR knock-out animals cannot be explained by our in vitro results. In our GWI model, we could not detect a significant difference for VSMC content between knock out and control animals nor could we find evidence for an uPAR mediated impact on VSMC proliferation. We cannot exclude that in other models or at a different time point uPAR deficiency might be more relevant for VSMC recruitment or proliferation.

Interestingly, in contrast to the diet-induced model of atherosclerosis, also other aspects of vascular remodelling in guide-wire-induced intimal hyperplasia in the carotid artery[17, 18] remained unaffected by the lack of uPAR. Neither plaque size, nor macrophage content were influenced. The reasons for this discrepancy have not been systematically investigated in this study. However, the interesting observations we made for uPAR deficient VSMCs in the context of vascular remodelling call for a separate, more detailed investigation. Guide-wire-induced intimal hyperplasia in hypercholesterolemic mice is driven by VSMC proliferation as well as monocyte recruitment.[28] In our experiments, attenuated monocyte adhesion was observed in vitro and likely accounts for the low macrophage content in the diet induced aortic sinus lesions. However, uPAR knock-out mice were not protected against adverse vascular remodelling after GWI. In this model monocyte recruitment might be less relevant as the endothelium in carotid arteries is denuded and effects other than endothelium dependent monocyte recruitment might come into play. However, also proliferation of VSMC seemed to be unaffected in vivo. In addition, the results of the guide-wire experiments might depend on the age of the mice. Our animals were started on the atherogenic diet at the age of six weeks. We cannot exclude that results might be different in older animals. Finally, lesions were only investigated four weeks after GWI. It is possible, that differences could have been uncovered at a different time point.

To corroborate our finding that uPAR specifically contributes to macrophage driven atherosclerotic lesion formation and to test its utility for therapeutic intervention we established a gene therapeutic strategy. We used hepatic overexpression of soluble full-length murine uPAR in Western-diet fed LDLR^-/-^ mice acting as a decoy receptor for uPAR ligands and competing endogenous membrane bound uPAR. This intervention reproduced findings pertaining to lesion size and monocyte recruitment that we made in the knockout animals. However, while the complete lack of uPAR protein led to reduced VSMC plaque content this was not observed in our gene therapeutic intervention. This observation indicates that with hepatic overexpression of soluble uPAR the signalling via endogenous membrane-bound uPAR is not fully silenced. Remaining endogenous uPAR activity thus explains the lack of effect on VSMC content after gene therapy.

In conclusion, both uPAR-deficiency and soluble full-length murine uPAR overexpressed in the liver and acting as a decoy receptor protect against atherosclerosis development in LDLR^-/-^ mice. These findings highlight the crucial role of uPAR in facilitating hyperlipidemia-induced plaque formation due to augmented lesional macrophage recruitment in addition to the recently postulated mechanisms underlying adverse effects of uPAR during atherogenesis.[7] In contrast, uPAR had no effect on neointimal hyperplasia after GWI of carotid arteries. Thus, uPAR may represent a promising therapeutic target to reduce hyperlipidemia-associated atherosclerotic lesion formation.

## Supporting Information

S1 Dataset Dataset. (XLSX)

## References

[1] Weber C, Noels H. Atherosclerosis: current pathogenesis and therapeutic options. Nat Med. 2011;17(11):1410–22. Epub 2011/11/09. doi: nm.2538 [pii] 10.1038/nm.2538 . 22064431

[2] Moore KJ, Sheedy FJ, Fisher EA. Macrophages in atherosclerosis: a dynamic balance. Nature reviews Immunology. 2013;13(10):709–21. 10.1038/nri3520 23995626 PMC4357520

[3] Shah PK, Falk E, Badimon JJ, Fernandez-Ortiz A, Mailhac A, Villareal-Levy G, et al Human monocyte-derived macrophages induce collagen breakdown in fibrous caps of atherosclerotic plaques. Potential role of matrix-degrading metalloproteinases and implications for plaque rupture. Circulation. 1995;92(6):1565–9. Epub 1995/09/15. . 7664441

[4] Blasi F, Carmeliet P. uPAR: a versatile signalling orchestrator. Nat Rev Mol Cell Biol. 2002;3(12):932–43. . 12461559 10.1038/nrm977

[5] Finn AV, Nakano M, Narula J, Kolodgie FD, Virmani R. Concept of vulnerable/unstable plaque. Arterioscler Thromb Vasc Biol. 2010;30(7):1282–92. Epub 2010/06/18. doi: 30/7/1282 [pii] 10.1161/ATVBAHA.108.179739 . 20554950

[6] Galis ZS, Sukhova GK, Lark MW, Libby P. Increased expression of matrix metalloproteinases and matrix degrading activity in vulnerable regions of human atherosclerotic plaques. The Journal of clinical investigation. 1994;94(6):2493–503. Epub 1994/12/01. 10.1172/JCI117619 7989608 PMC330083

[7] Farris SD, Hu JH, Krishnan R, Emery I, Chu T, Du L, et al Mechanisms of urokinase plasminogen activator (uPA)-mediated atherosclerosis: role of the uPA receptor and S100A8/A9 proteins. J Biol Chem. 2011;286(25):22665–77. Epub 2011/05/04. 10.1074/jbc.M110.202135 21536666 PMC3121410

[8] Smith HW, Marshall CJ. Regulation of cell signalling by uPAR. Nature reviews Molecular cell biology. 2010;11(1):23–36. Epub 2009/12/23. 10.1038/nrm2821 . 20027185

[9] May AE, Kanse SM, Lund LR, Gisler RH, Imhof BA, Preissner KT. Urokinase receptor (CD87) regulates leukocyte recruitment via beta 2 integrins in vivo. J Exp Med. 1998;188(6):1029–37. . 9743521 10.1084/jem.188.6.1029 PMC2212528

[10] Larmann J, Frenzel T, Hahnenkamp A, Herzog C, Lorenz A, Steinbicker AU, et al In Vivo Fluorescence-mediated Tomography for Quantification of Urokinase Receptor-dependent Leukocyte Trafficking in Inflammation. Anesthesiology. 2010;113(3):610–8. Epub 2010/08/10. 10.1097/ALN.0b013e3181e99bfc . 20693875

[11] Steins MB, Padro T, Schwaenen C, Ruiz S, Mesters RM, Berdel WE, et al Overexpression of urokinase receptor and cell surface urokinase-type plasminogen activator in the human vessel wall with different types of atherosclerotic lesions. Blood coagulation & fibrinolysis: an international journal in haemostasis and thrombosis. 2004;15(5):383–91. Epub 2004/06/19. . 15205586 10.1097/01.mbc.0000114441.59147.56

[12] Svensson PA, Olson FJ, Hagg DA, Ryndel M, Wiklund O, Karlstrom L, et al Urokinase-type plasminogen activator receptor is associated with macrophages and plaque rupture in symptomatic carotid atherosclerosis. International journal of molecular medicine. 2008;22(4):459–64. Epub 2008/09/25. . 18813852

[13] Chen W, Jin WQ, Chen LF, Williams T, Zhu WL, Fang Q. Urokinase receptor surface expression regulates monocyte migration and is associated with accelerated atherosclerosis. International journal of cardiology. 2012;161(2):103–10. Epub 2012/01/13. 10.1016/j.ijcard.2011.12.094 . 22236514

[14] Anaraki PK, Patecki M, Larmann J, Tkachuk S, Jurk K, Haller H, et al Urokinase Receptor Mediates Osteogenic Differentiation of Mesenchymal Stem Cells and Vascular Calcification via the Complement C5a Receptor. Stem cells and development. 2014;23(4):352–62. Epub 2013/11/07. 10.1089/scd.2013.0318 24192237 PMC3920849

[15] Ishibashi S, Goldstein JL, Brown MS, Herz J, Burns DK. Massive xanthomatosis and atherosclerosis in cholesterol-fed low density lipoprotein receptor-negative mice. The Journal of clinical investigation. 1994;93(5):1885–93. Epub 1994/05/01. 10.1172/JCI117179 8182121 PMC294295

[16] Dewerchin M, Nuffelen AV, Wallays G, Bouche A, Moons L, Carmeliet P, et al Generation and characterization of urokinase receptor-deficient mice. J Clin Invest. 1996;97(3):870–8. . 8609247 10.1172/JCI118489 PMC507128

[17] Lindner V, Fingerle J, Reidy MA. Mouse model of arterial injury. Circ Res. 1993;73(5):792–6. Epub 1993/11/01. . 8403250 10.1161/01.res.73.5.792

[18] Quarck R, De Geest B, Stengel D, Mertens A, Lox M, Theilmeier G, et al Adenovirus-mediated gene transfer of human platelet-activating factor-acetylhydrolase prevents injury-induced neointima formation and reduces spontaneous atherosclerosis in apolipoprotein E-deficient mice. Circulation. 2001;103(20):2495–500. Epub 2001/05/23. . 11369691 10.1161/01.cir.103.20.2495

[19] De Geest B, Zhao Z, Collen D, Holvoet P. Effects of adenovirus-mediated human apo A-I gene transfer on neointima formation after endothelial denudation in apo E-deficient mice. Circulation. 1997;96(12):4349–56. Epub 1998/01/07. . 9416903 10.1161/01.cir.96.12.4349

[20] Ye X, Loeb KR, Stafford DW, Thompson AR, Miao CH. Complete and sustained phenotypic correction of hemophilia B in mice following hepatic gene transfer of a high-expressing human factor IX plasmid. J Thromb Haemost. 2003;1(1):103–11. Epub 2003/07/23. doi: 24 [pii]. . 12871546 10.1046/j.1538-7836.2003.00024.x

[21] Vorup-Jensen T, Jensen UB, Liu H, Kawasaki T, Uemura K, Thiel S, et al Tail-vein injection of mannan-binding lectin DNA leads to high expression levels of multimeric protein in liver. Mol Ther. 2001;3(6):867–74. Epub 2001/06/16. 10.1006/mthe.2001.0335 S1525-0016(01)90335-1 [pii]. . 11407900

[22] Larmann J, Schmidt C, Gammelin H, Van Aken HK, Frenzel T, Lanckohr C, et al Intercellular adhesion molecule-1 inhibition attenuates neurologic and hepatic damage after resuscitation in mice. Anesthesiology. 2005;103(6):1149–55. . 16306726 10.1097/00000542-200512000-00008

[23] Theilmeier G, Schmidt C, Herrmann J, Keul P, Schafers M, Herrgott I, et al High-density lipoproteins and their constituent, sphingosine-1-phosphate, directly protect the heart against ischemia/reperfusion injury in vivo via the S1P3 lysophospholipid receptor. Circulation. 2006;114(13):1403–9. . 16982942 10.1161/CIRCULATIONAHA.105.607135

[24] Theilmeier G, Quarck R, Verhamme P, Bochaton-Piallat ML, Lox M, Bernar H, et al Hypercholesterolemia impairs vascular remodelling after porcine coronary angioplasty. Cardiovasc Res. 2002;55(2):385–95. Epub 2002/07/19. doi: S0008636302004091 [pii]. . 12123778 10.1016/s0008-6363(02)00409-1

[25] Kiyan J, Kusch A, Tkachuk S, Kramer J, Haller H, Dietz R, et al Rosuvastatin regulates vascular smooth muscle cell phenotypic modulation in vascular remodeling: role for the urokinase receptor. Atherosclerosis. 2007;195(2):254–61. Epub 2007/02/06. 10.1016/j.atherosclerosis.2006.12.030 . 17275828

[26] Kiyan Y, Tkachuk S, Hilfiker-Kleiner D, Haller H, Fuhrman B, Dumler I. oxLDL induces inflammatory responses in vascular smooth muscle cells via urokinase receptor association with CD36 and TLR4. J Mol Cell Cardiol. 2014;66:72–82. Epub 2013/11/19. 10.1016/j.yjmcc.2013.11.005 . 24239845

[27] Plekhanova O, Parfyonova Y, Bibilashvily R, Domogatskii S, Stepanova V, Gulba DC, et al Urokinase plasminogen activator augments cell proliferation and neointima formation in injured arteries via proteolytic mechanisms. Atherosclerosis. 2001;159(2):297–306. Epub 2001/12/04. . 11730809 10.1016/s0021-9150(01)00511-1

[28] Hui. Intimal Hyperplasia in Murine Models. Curr Drug Targets. 2008;9(3):251–60. 18336244 10.2174/138945008783755601 PMC2829189

